# Immune microenvironment characteristics in multiple myeloma progression from transcriptome profiling

**DOI:** 10.3389/fonc.2022.948548

**Published:** 2022-08-12

**Authors:** Jin Wang, Yi Hu, Habib Hamidi, Cedric Dos Santos, Jingyu Zhang, Elizabeth Punnoose, Wenjin Li

**Affiliations:** ^1^ Oncology Biomarker Development, Roche (China) Holding Ltd., Shanghai, China; ^2^ Oncology Biomarker Development, Genentech, Ltd., South San Francisco, CA, United States

**Keywords:** multiple myeloma, microenvironment, transcriptome, immunotherapy, autologous stem cell transplantation (ASCT)

## Abstract

Multiple myeloma (MM) is characterized by clonal expansion of malignant plasma cells in the bone marrow (BM). Despite the significant advances in treatment, relapsed and refractory MM has not yet been completely cured due to the immune dysfunction in the tumor microenvironment (TME). In this study, we analyzed the transcriptome data from patients with newly diagnosed (ND) and relapsed/refractory (R/R) MM to characterize differences in the TME and further decipher the mechanism of tumor progression in MM. We observed highly expressed cancer testis antigens and immune suppressive cell infiltration, such as Th2 and M2 cells, are associated with MM progression. Furthermore, the TGF-β signature contributes to the worse outcome of patients with R/R MM. Moreover, patients with ND MM could be classified into immune-low and immune-high phenotypes. Immune-high patients with higher IFN-g signatures are associated with MHC-II–mediated CD4+ T-cell response through CIITA stimulation. The baseline TME status could potentially inform new therapeutic choices for the ND MM who are ineligible for autologous stem cell transplantation and may help predict the response to CAR-T for patients with R/R MM. Our study demonstrates how integrating tumor transcriptome and clinical information to characterize MM immune microenvironment and elucidate potential mechanisms of tumor progression and immune evasion, which will provide insights into MM treatment selection.

## Introduction

Multiple myeloma (MM) is a malignant tumor of terminally differentiated plasma cells (PCs), which accounts for about 10% of all hematological malignancies (1). It is the second most common hematological malignancy after non-Hodgkin’s lymphoma. In the United States, more than 32,000 new cases are diagnosed each year, nearly 13,000 of which die from it (2). MM remains incurable despite advances in treatment, with an estimated median survival of 8–10 years for standard-risk myeloma and 2–3 years for high-risk disease, and despite consolidation of response with an autologous stem cell transplant (3). Only 10%–15% of patients achieve or exceed expected survival compared with the matched general population (4). There is no single standard of care for relapsed and refractory MM, and treatment is affected by several factors, including age, performance status, comorbidities, and the type, efficacy, and tolerance of the previous treatments, underlying a strong unmet need for novel treatments for patients with relapsed and refractory disease.

A total of 16,500 new cases and 10,300 deaths of MM were reported in China in 2016. The age-standardized incidence rates and mortality rates were 1.03 and 0.67 per 100,000 population, respectively (5). There is variation in incidence among the different races: African Americans have twice the incidence of myeloma as whites. In contrast, Asians have a relatively low incidence (6).

Generally, most patients with MM are characterized by the secretion of monoclonal immunoglobulin protein, which is produced by abnormal PCs within the bone marrow (BM) (7). The clinical outcome of MM is primarily driven by acquired genetic factors and changes within the tumor microenvironment (TME) (8). Structural mutations, such as chromosomal translocations and copy number variations (CNVs), ultimately promote relapse and disease progression (9, 10). Existing studies have reported that MM can be broadly split into cases with oncogenic Ig translocations and those that are hyperdiploid with odd number chromosomes. The most frequent translocations are the t(11;14), t(4;14), t(14;16), t(14;20), and t(6;14), and additional CNVs occur with the most frequent being del13q, 1q+, del14q, del6q, del1p, and del17p (10). Associated with the evidence, the mutations in 63 driver genes related to the progression of MM have been identified, including IDH1/2, HUWE1, KLHL6, and PTPN11 (11). The frequent chromosomal translocations can also lead to gene fusions, such as MYC-PVT1 fusion (12), which will significantly impact both progression-free and overall survival, and even act as drivers of MM (13).

Despite the significant therapeutic progress with the great effort on the explorations in these oncogenes over the last decade, active MM remains an incurable disease due to the immune dysfunction and immune suppression in the TME (14). Although various immunotherapies have been applied to block the proliferation of malignant PCs (15), avoiding immune evasion in active MM progression still remains a challenge (16). With the vital role of the TME in myeloma development, previous studies have shown that immune cell infiltration of TME is closely related to the prognosis and malignancy of tumors (17). Clarifying the relationship between the immune microenvironment and the progression of MM cancer is conducive to improve the effect of treatment. Furthermore, the therapeutic effect is restricted by the TME heterogeneity, which dynamically changes with distinct immune cell infiltration and immune mediator profiling (18). As a result, the traditional and novel MM therapies remain uncertain (19). However, the related transcriptome analysis on MM cohorts is limited now. Therefore, characterizing the TME in MM from transcriptome profiling has a decisive impact to improve treatment efficacy in clinical practice.

Here, we characterized the TME differences between patients with newly diagnosed (ND) and relapsed/refractory (R/R) MM, which represent the primary and recurrent MM in this public data set, with comprehensive transcriptome analysis to elucidate the potential mechanism of MM progression. In addition, we also described the immune status of patients with ND MM to interpret the potential relationship between baseline TME and treatment efficacy, potentially providing a new avenue of improving clinical treatment by remodeling TME.

## Materials and methods

### Data collection and preprocessing

We collected transcriptome data from MMRF (The Multiple Myeloma Research Foundation) with 787 patients and 859 samples, which is available at https://portal.gdc.cancer.gov/. These samples are collected from either peripheral blood or BM in patients with ND and R/R MM along with clinical data, including line therapy, drug treatment, and overall survival information (**Figures S1A, B**, **Supplementary Table S1**). Given the comparable data size, all the samples from BM with 764 ND and 80 R/R MM (**Figure S1A**, **Supplementary Table S2**), which were CD138 enriched, were retained for all the downstream analysis. In addition, as for patients with R/R MM, some of them have samples collected after more than one treatment, and there was redundancy at the sample level, which was all included in the downstream analysis. HTSeq processed read count data were downloaded and converted to transcript per million (TPM) for gene quantification. The annotation gtf file was downloaded from Gencode website on version 22 of hg38 (https://www.gencodegenes.org/human/), which is used to transfer Ensembl ID to gene symbol.

### Differential expression and gene set enrichment analysis

Differential expression analysis on 764 ND and 80 R/R samples was performed using DESeq2 with read count data from HT-Seq quantification (20). Downstream gene set enrichment analysis was performed by GSEA using the pre-ranked genes by fold change from output of DESeq2 (21). Gene sets are collected from the Molecular Signatures Database (MSigDB v7.1; 34), including 186 Kyoto Encyclopedia of Genes and Genomes (KEGG) pathways (22) and 50 hallmark pathways. KEGG pathway categories are referred from KEGG website (https://www.genome.jp/kegg/pathway.html). Significant upregulated and downregulated pathways [false discovery rate (FDR) ≤ 0.05] were identified. This analysis was fulfilled by GSEA v4.0.3 for Linux, and figures were generated by the ComplexHeatmap R package v1.99.5.

### Immune infiltration estimation from RNA-seq data

To ensure robust abundance estimation, we applied xCell to conduct deconvolution (11) of immune cell components from log2-transformed TPM expression because xCell covers much more cell types than the other algorithms (**Supplementary Table S3**).

### The immune signature score for classifying “immune-low” and “immune-high” MM tumors

From the pan-cancer paper (23), the immune signature data and the pre-trained scoring model were downloaded from the CRI-iAtlas (https://github.com/CRI-iAtlas/shiny-iatlas/), which integrated five representative immune signatures trained from The Cancer Genome Atlas (TCGA) Pan-cancer profilings. On the basis of the model, we computed the five representative immune signatures scores from the expression data (TPM) normalized by the gene-wise median-centered value. These signatures include “IFN-g” (representing IFN-g response), “Lymphocyte” (representing overall lymphocyte infiltration by B and T cells), “Macrophage” (representing the activation of macrophages/monocytes), “TGF-β” (representing TGF-β response), and “Wound healing” (representing core serum response). Then, we performed hierarchical clustering on the MM tumors with the representative immune signature scores into two groups, which are defined as immune-low and immune-high.

### Statistical analysis

For all the statistical tests comparing the difference between tumor and adjacent tissues, Mann–Whitney U-test was used to evaluate the P-values, and P < 0.05 was considered statistically significant. Multiple hypothesis correction was computed in the form of adjusted p-value through the Benjamini–Hochberg procedure. Spearman’s rank correlation coefficient was presented as the association of two independent features. All the statistical tests were implemented using the R programming language.

## Results

### Highly expressed cancer testis antigens and immune suppressive cell infiltration are associated with MM cancer progression

**Figure 1:**
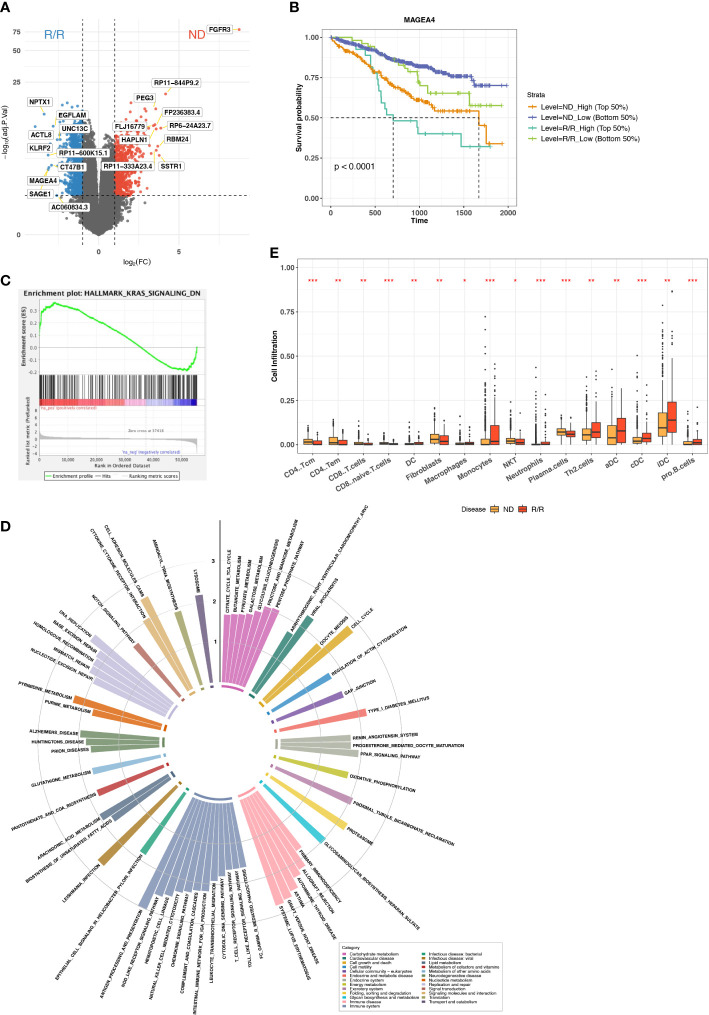
Immune microenvironment difference between ND and R/R MMs. **(A)** Differential expression analysis between ND and R/R MM tumors. Red dots indicate the top 10 upregulated genes in ND MM tumors, whereas blue dots indicate top 10 upregulated genes in R/R MM tumors. **(B)** Kaplan–Meier survival curve for MAGEA4 expression. **(C)** Enrichment plot from GSEA for KRAS signaling downregulation in ND MM tumors. **(D)** Normalized enrichment scores of tumor-upregulated KEGG pathways. Colors indicate different KEGG pathway categories. Asterisks denote significance level (*** FDR < 0.001; ** FDR < 0.01; * FDR < 0.05; NS FDR > 0.05). **(E)** Comparison of Cell infiltration estimated from xCell between ND and R/R MM tumors. Asterisks denote significance level (*** P < 0.001; ** P < 0.01; * P < 0.05; NS P > 0.05).

We collected transcriptome data from MMRF (MMRF-COMPASS) with 787 patients and 859 samples at ND or R/R from either BM or peripheral blood (**Figure S1A**). To keep the consistent tissue source and comparable sample size, we only retained the 844 samples from BM. Because the mechanism of myeloma progression or immune evasion still remains unclear, we sought to characterize the immune microenvironment landscape from transcriptome. We performed standard differential expression analysis using DESeq2 to identify the genes specifically expressed in ND or R/R (20). In total, we identified 998 upregulated genes and 815 downregulated genes in R/R MM samples with over two-fold change and a FDR of less than 0.05. In particular, we observed cancer testis antigen (CTA) genes were the most differentially expressed genes in R/R MM samples, including ACTL8, SAGE1, MAGEA4, and CT47B1 (**Figure 1A**). CTA genes have been reported to be highly expressed in testis and atypically expressed in about 40% of different types of cancers (24). Previous studies have revealed CTA genes as prognostic markers and potential targets in immunotherapy (25). Furthermore, overexpression of CTA genes in R/R MM samples was associated with worse outcome (**Figures 1B**, **S2A**).

To examine the altered pathways driven in R/R MM samples based on the comparison between baseline and R/R samples, we then applied GSEA (21) based on KEGG (22) and cancer Hallmark pathways (26). The vast majority of Hallmark signals were enriched in R/R MMs (**Figure S2B**), suggesting the reaction response during MM progression. In addition to cancer Hallmark pathways, KRAS signaling is upregulated in R/R MMs but downregulated in ND MMs (**Figure 1C**), indicating KRAS-mediated oncogenic signaling activation in MM progression (27). Moreover, the pathways related to immune disease and immune systems were enriched in R/R MM samples, such as antigen processing and pathway, presentation, T-cell receptor signaling pathway, and natural killer (NK) cell–mediated cytotoxicity (**Figure 1D**). Here, Major histocompatibility complex (MHC) molecules were highly expressed in R/R MM samples, whereas MHC-I molecules were not differentially expressed (**Figure S2C**), suggesting that cancer cells escaped CD8+ T-cell–mediated cytotoxic killing but not NK cell–mediated cytotoxic killing and MHC-II–mediated CD4+ T-cell activation. This motivated us to do further exploration on immune cell infiltration in the MM microenvironment. Indeed, CD8+ T cells were not highly enriched in R/R MM samples, whereas CD4+ T-cell–differentiated Th2 cells were highly enriched in R/R MMs from xCell (28) estimation (**Figure 1E**), which will contribute to the immune suppressive MM microenvironment. We further examined the difference of M1/M2 ratio between ND and R/R MM. Moreover, R/R MMs was of higher fraction of M2 macrophages than ND MMs by colony-stimulating factor 1 (CSF1) enhancing M2 polarization (29) (**Figures S2D, E**), contributing to immune suppressive phenotype for MM progression. This is consistent with recent findings that M2 polarization may play a functional role in MM disease progression (30, 31) and further supported that targeting macrophages, *via* CSF1 receptor, in combination with standard treatment modalities, may be a promising therapeutic strategy in MM. Collectively, overexpression of CTA genes and immune suppressive cell infiltration are associated with MM cancer progression.

### TGF-β response signature and abnormal plasma cell accumulation are unfavorable features for R/R MM outcomes

**Figure 2:**
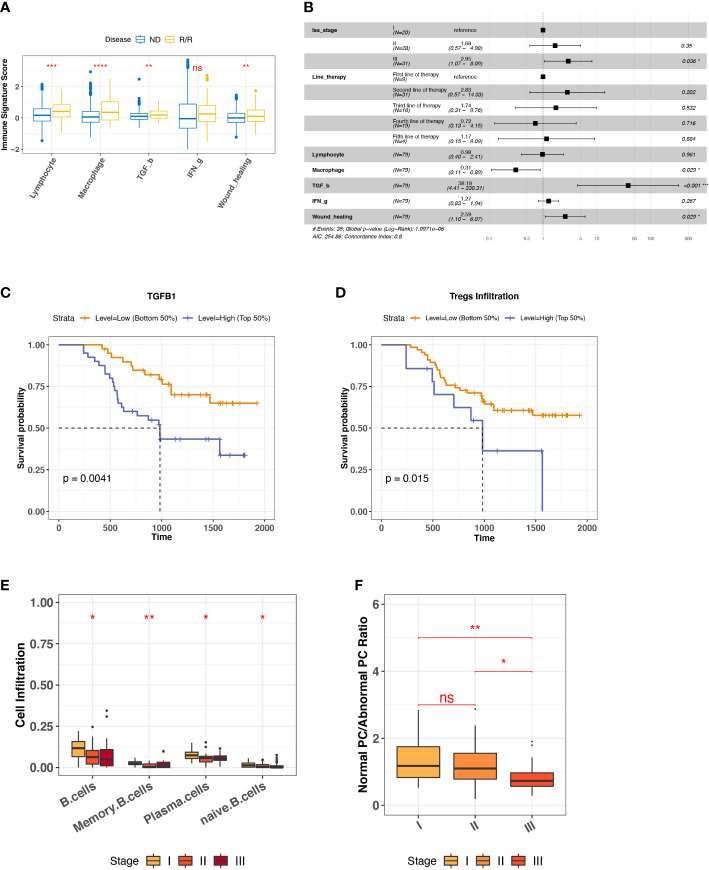
Biomarkers for R/R MM prognosis. **(A)** Comparison of representative immune signature score between ND and R/R MM tumors. Asterisks denote significance level (**** P < 0.0001; *** P < 0.001; ** P < 0.01; * P < 0.05; NS, not significant, P > 0.05). **(B)** Forest plot for cox proportional hazard model with representative immune signatures corrected by line therapy and tumor stage. **(C)** Kaplan–Meier survival curve for TGFB1 expression. Two groups are defined by median expression of TGFB1. **(D)** Kaplan–Meier survival curve for Treg cell infiltration. Two groups are defined by median infiltration level of Treg cells. **(E)** B-cell lineage infiltration comparison across different tumor stages. Asterisks denote significance level (*** P < 0.001; ** P < 0.01; * P < 0.05; NS P > 0.05). **(F)** Ratio of normal PC to abnormal PC comparison across different tumor stages. Asterisks denote significance level (*** P < 0.001; ** P < 0.01; * P < 0.05; NS P > 0.05). ns, Not significant.

To characterize the overall immune microenvironment in MM, we used five representative immune signatures based on a TCGA pre-trained model (23) to compare immune signature scores between ND and R/R MM samples. Consequently, R/R MMs were found having a higher level of most representative immune signatures, including Lymphocyte, Macrophage, TGF-β, and Wound healing (**Figure 2A**). In particular, TGF-β response signature is an unfavorable feature for R/R MMs from a proportional hazards model corrected by the confounding factors, such as tumor stage and line therapy (**Figure 2B**). Consistently, R/R MMs with higher TGF-β response signature score or higher expression of TGFB are associated with poor prognosis (**Figures 2C**, **S3A**). In keeping with the fact that regulatory T (Treg) cells are one major source of secreting TGFB and that TGFB regulates the generation of Treg cells in turn (32), the R/R MM samples with higher Treg cells were also associated with worse outcome (**Figure 2D**). In addition to TGFB and Tregs cells, we also found higher expression of T-cell exhaustion marker CTLA4 and TIM3 (HAVCR2) in R/R MM tumors than ND tumors (P-value < 0.01) (**Figure S3B**). In particular, high expression of CTLA4 was observed to be associated with worse outcome (P-value = 0.036) (**Figure S3C**), indicating a potential biomarker of R/R MM tumor prognosis.

To investigate the factors influencing R/R MM tumor (N = 80) progression, we checked the distinct cell infiltration in different tumor stages. We observed the significant infiltration differences among B-cell lineage, such as the decreased naïve B cells and normal PCs in the later stage (**Figure 2E**), the low infiltration of which was associated with worse outcome (**Figure S3D**). It is well known that MM is caused by abnormal PC generation during B-cell maturation. To quantify the degree of normal PCs over myeloma PCs during MM progression, we measured the ratio of normal PC to abnormal PC by the averaged TPM values of three phenotype markers, respectively. Normal PCs are represented by CD19, CD27, and CD81, whereas abnormal PC are represented by CD20, CD56, and CD117 (33, 34). It provided the evidence of abnormal PC generation accumulated during MM progression (**Figure 2F**). Taken together, we revealed that TGF-β response signature and abnormal PC accumulation are unfavorable features for R/R MM outcomes.

### Immune-high ND MMs with higher IFN-g signatures stimulate CIITA expression to induce MHC-II–mediated CD4+ T-cell response

**Figure 3:**
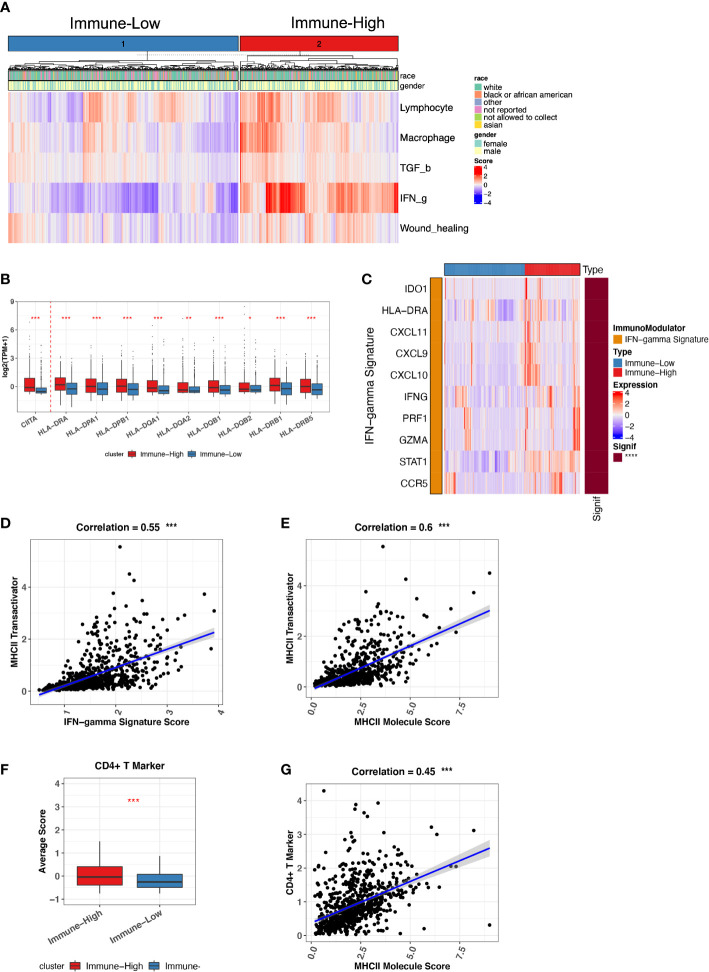
Immune-low and Immune-high ND MM tumor classification based on representative immune signatures. **(A)** ND MM tumors (x-axis) clustered by representative immune signature score (heatmap colors). The top bar shows race and gender information for each patient. **(B)** Comparison of CIITA and MHC-II molecules expression between immune-low and immune-high tumors. Asterisks denote significance level (*** P < 0.001; ** P < 0.01; * P < 0.05; NS P > 0.05). **(C)** Expression of IFN-g signature genes in immune-low and immune-high tumors. The right bar shows the significance of gene difference between immune-low and immune-high tumors. **(D)** The correlation between IFN-g signature score and CIITA expression. **(E)** The correlation between MHC-II molecules expression and CIITA expression. **(F)** Average score difference of CD4+ T-cell markers between immune-low and immune-high tumors. **(G)** The correlation between MHC-II molecules expression and CD4+ T-cell marker score.

Because we have a large size of ND MM BMA samples (764 ND MMs), we sought to investigate ND MMs subgroups to classify different immune status. We differentiated ND MMs based on the overall immune microenvironment by the five representative immune signatures, finally resulting in immune-high and immune-low clusters (**Figure 3A**). In particular, immune-high samples have stronger IFN-g response signature score compared with immune-low samples, potentially playing the role in immune surveillance or immune evasion (33). Thus, it is essential to decipher the effect of high IFN-g response in Immune-high ND MM samples. In addition to the high level of IFN-g response, we also observed that the immune-high ND MM samples have higher MHC-II molecules expression (**Figure 3B**). It has been reported that IFN-g signatures stimulate CIITA expression to induce MHC-II–mediated CD4+ T-cell response. Immune-high ND MM samples highly expressed CIITA (**Figure 3B**), known as MHC-II transactivator and upregulate the expression of MHC-II molecules (35). Therefore, we next investigated the interaction of IFN-g signature score, CIITA expression, MHC-II level, and CD4+ T-cell markers. First, we checked the IFN-g signature related genes, including IDO1, HLA-DRA, CXCL11, CXCL9, CXCL10, IFNG, PRF1, GZMA, STAT1, and CCR5 (36). The samples in immune-high ND MM cluster had higher expression of IFN-g signature score, which was defined as the averaged expression of the above IFN-g signature genes (**Figure 3C**). Furthermore, the positive association between IFN-g signature score and CIITA indicated the potential stimulation of IFN-g signatures on CIITA (**Figure 3D**). Next, we observed CIITA was associated with MHC-II molecule expression, suggesting MHC-II production induced by CIITTA (**Figure 3E**). To quantify CD4+ T-cell response, we measured CD4+ T-cell response by CD4+ T-cell markers (CD3D, CD3E, CD4, and CD40LG), which was also highly expressed in immune-high ND MM samples (**Figure 3F**) and significantly associated with MHC-II expression (**Figure 3G**). Moreover, Th1 and Th2 cells differentiated from CD4+ T cells were highly infiltrated in immune-high ND MM samples (**Figure S4A**), demonstrating higher CD4+ T-cell response in immune-high ND MM samples. Moreover, the high infiltration of Th2 was associated with worse outcome (**Figure S4B**), which was consistent with the suppressive immune phenotype of Th2. Collectively, these observations revealed distinct immune phenotypes in ND MM samples and the interactions between IFN-g signatures expression, CIITA expression, MHC-II level, and CD4+ T-cell response in immune-high ND MM samples.

### Baseline immune microenvironment can inform therapeutic choice for ASCT-ineligible ND MM and R/R MM

**Figure 4:**
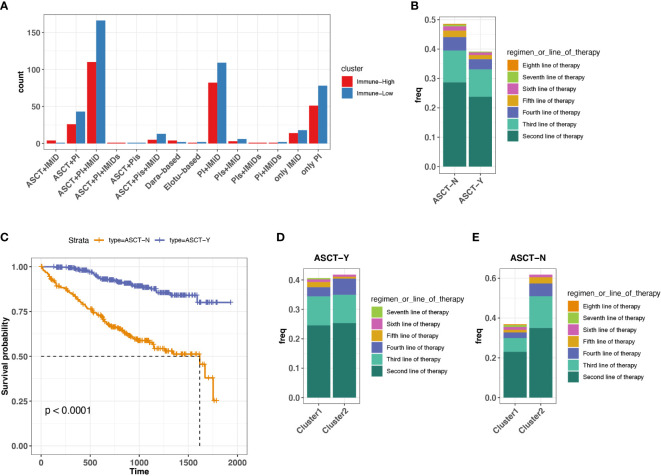
Association between treatment selection immune microenvironment in ND MM tumors. **(A)** Patient count with different drug combos in immune-low (cluster 1) and immune-high (cluster 2) tumors. **(B)** Frequency receiving treatments after the first-line therapy for the patients with or without prior ASCT treatment. Colors indicate different line therapies. **(C)** Kaplan–Meier survival curve for ASCT treatment. **(D)** Comparison of frequency receiving treatments after the first-line therapy between immune-low (cluster 1) and immune-high (cluster2) MM tumors with ASCT. Colors indicate different line therapies. **(E)** Comparison of frequency receiving treatments after the first-line therapy between immune-low (cluster 1) and immune-high (cluster2) MM tumors without ASCT. Colors indicate different line therapies.

On the basis of the immune-high and immune-low subgroups that we identified in ND MMs, we explored the potential relationship between immune microenvironment and treatment selection. In this cohort, the patients were treated with protein inhibitor (PI), immunomodulatory imide drugs (IMid), and PI plus IMid, with or without autologous stem cell transplant (ASCT) (**Figure 4A**). ASCT remains the standard of care for patients with ND MM (37). Therefore, we grouped the samples based on ASCT treatment due to the balanced size. We found that the patients with ASCT have lower probability of receiving treatments after first-line therapy compared with patients without ASCT (**Figure 4B**). Furthermore, the patients with ASCT gained better prognosis (**Figure 4C**). Moreover, the patients with ASCT have the similar probability of receiving treatments after First-line therapy regardless of immune-high or immune-low microenvironment (**Figure 4D**). However, the patients without ASCT of immune-high microenvironment have higher chance of receiving treatments after first-line therapy compared with that of immune-low microenvironment (**Figure 4E**), suggesting the influence of baseline immune system function on MM tumor treatment without ASCT. Consequently, for ASCT-ineligible patients with ND MM, who have a worse prognosis compared with those ASCT-eligible, once their baseline TME was classified as immune-high, they received more treatments than those with immune-low TME.

We further attempt to explore new therapies for patients not amenable to ASCT in ND MM. With numerous immunomodulators (IMs) agonists and antagonists being evaluated in clinic, understanding the expression of IMs in different states of the MM immune microenvironment is critical for cancer immunotherapy selections. Current IMs cover targets, playing a role in multiple functions, such as receptor, co-inhibitor, ligand, co-stimulator, antigen presentation, and cell adhesion (23). We found higher expression of these IMs in patients with MM with immune-high microenvironment compared with those with immune-low microenvironment, such as the common immune checkpoint inhibitors (PD1, CTLA4, LAG3, TIGIT, TIM3, and BTLA) (34) (**Figure S5**). In addition, SLAMF7, a robust marker of malignant PCs in MM tumors (38, 39), was highly expressed in immune-high MM tumors, which has been regarded as a target for immunotherapy (Elotuzumab). VEGF (known as vascular endothelial growth factor) has been reported to induce modest proliferation of MM cells (40), also highly expressed in immune-high MM tumors. IM targets potentially open the new avenue for the treatments on patients who are not eligible for ASCT. Taken together, treatments targeting IMs might be alternative for the ASCT-ineligible patients with ND MM with baseline immune-high status.

Moreover, we examined the IM expression difference in immune-high and immune-low groups of R/R MM, which showed similar results with what we found in ND MM (**Figure S6**). Because CAR T-cell therapy has been FDA-approved for the treatment of R/R MM, and multiple reports indicated that high level expression of IMs like PD1, LAG3, and TIM3 and their receptors were associated with T-cell exhaustion and poor response to CAR-T therapy in acute lymphoblastic leukemia (41, 42), probably R/R MM with immune-low status is more recommend to receive CAR-T therapy. Consequently, immunophenotyping classified by immune-high or immune-low might also have clinical impact for patients with R/R MM.

## Discussion

In this study, we characterized the immune microenvironment of MM tumors by integrating transcriptome data (molecular profiling and immune cell estimation) and clinical information (overall survival and line therapy). Our observation of highly expressed CTA genes and active immune-related pathways in R/R MM tumors described MM tumor evolution. From immune cell estimation, we observed tumor-infiltrating M2 polarization was dominated, and CD4+ T-cell tended to differentiate with Th2 cells in R/R MM tumors, both of which contributed to the suppressive immune microenvironment during MM tumor progression. The observations confirmed the reports in previous study for immune evasion and the loss of immune surveillance in MM tumors (43, 44). To investigate the features associated with R/R MM survival, we characterized the overall immune status of tumors with five representative immune signatures and found the high level of TGF-β signatures associated with worse outcome in R/R MM tumors. The results were corroborated using both of TGF-β expression and TGF-β secreting Treg cell infiltration. T-cell exhaustion markers, CTLA4 and TIM3, were highly expressed in R/R tumors and associated with worse prognosis. These biomarkers characterized unfavorable features in R/R MM prognosis.

In addition, we also observed the decreased normal B-cell lineage and the accumulated aberrant PCs in late R/R tumor stage. Moreover, we classified ND MM tumors into immune-low and immune-high tumors based on the overall immune status. Immune-high tumors were found to have higher IFN-g signature, which was strongly associated with CIITA expression to stimulate MHC-II generation helping CD4+ T-cell activation and differentiation. Potentially, immune-high MM tumors that were ineligible for ASCT tended to receive treatments after the first-line therapy. However, these tumors expressed higher levels of IMs, which will open new avenues for the treatment of MM tumors ineligible for ASCT.

Previous studies have reported some factors associated with immune dysfunction and evasion to characterize MM tumor evolution and disease progression, such as B-cell dysfunction (45), deficiencies in T-cell function (46, 47), and upregulation of immune suppressive cytokines (47). Currently, our analysis from transcriptome profiling comprehensively revealed the major immune cell infiltration and cytokine secretion associated with MM evolution. In particular, we highlighted the reduction of CD8+ T-cell cytotoxicity, the enhancement of CD4+ T-cell differentiation, and the polarization of M2 phenotype during MM progression. Moreover, TGF-β signature and aberrant PC accumulation were unfavorable features for R/R MM patient outcomes. In addition to the tumor progression mechanism, the treatment ways of restoring adaptive immunity in myeloma are also essential to improve MM tumor responses. New therapeutic opportunities, such as immunomodulatory agents, immune checkpoint blockade, chimeric antigen receptor T cells, bispecific T-cell engagers, and anti-MM vaccination, have been established to become promising novel options for patients with MM. Nevertheless, effective treatments for MM tumors remain lacking due to the complexity and heterogeneity of MM immune microenvironment. The treatments vary for patients with distinct immune microenvironment. Hence, we stratified MM tumors based on immune microenvironment features into immune-low and immune-high clusters. Immune-high tumors had strong IFN-g signature to induce MHC-II–mediated CD4+ T-cell response for promoting Th2 cell differentiation, which was associated with an immune suppressive environment and worse prognosis. The patients in immune-high status and ineligible for ASCT potentially benefit from the IMs with high expression level because ASCT has remodeled the immune system functions. In addition, the baseline immune status has high potential to help predict the response to CAR-T therapy in R/R MM. We were the first to explore the TME of MMRF cohorts by profiling the transcriptome. These observations characterized the features involved in MM tumor progression and provide insights into MM treatment for patients with distinct immune microenvironment. However, additional research and clinical studies are required to fully elucidate the mechanism of MM evolution and explore better immunotherapy strategies in MM cancer.

## Data availability statement

The original contributions presented in the study are included in the article/**Supplementary Material**. Further inquiries can be directed to the corresponding author.

## Author contributions

Conception/design: WL and JW. Data sorting and assembly: JW. Data analysis and interpretation: JW, WL, YH, HH, CS, JZ, and EP. Manuscript writing: WL, JW, and YH. Final approval of manuscript: JW, YH, HH, CS, JZ, EP, and WL. All authors contributed to the article and approved the submitted version.

## Funding

This work was funded by F. Hoffmann–La Roche/Genentech.

## Conflict of interests

JW, YH, JZ, and WL are employed by Roche (China) Holding, Ltd. HH, CS, and EP are employed by Genentech, Ltd.

All the authors declare that the research was conducted in the absence of any further commercial or financial relationships that could be construed as a potential conflict of interest.

## Publisher’s note

All claims expressed in this article are solely those of the authors and do not necessarily represent those of their affiliated organizations, or those of the publisher, the editors and the reviewers. Any product that may be evaluated in this article, or claim that may be made by its manufacturer, is not guaranteed or endorsed by the publisher.
