# Supplementary material for: Immune microenvironment characteristics in multiple myeloma progression from transcriptome profiling
Source: Front Oncol. 2022 Aug 12;12:948548. doi: 10.3389/fonc.2022.948548 (PMC9413314; doi:10.3389/fonc.2022.948548)
Supplement: Supplementary file 1 [file DataSheet_1.pdf]

Supplementary Material

1 Supplementary Figures

Figure S1

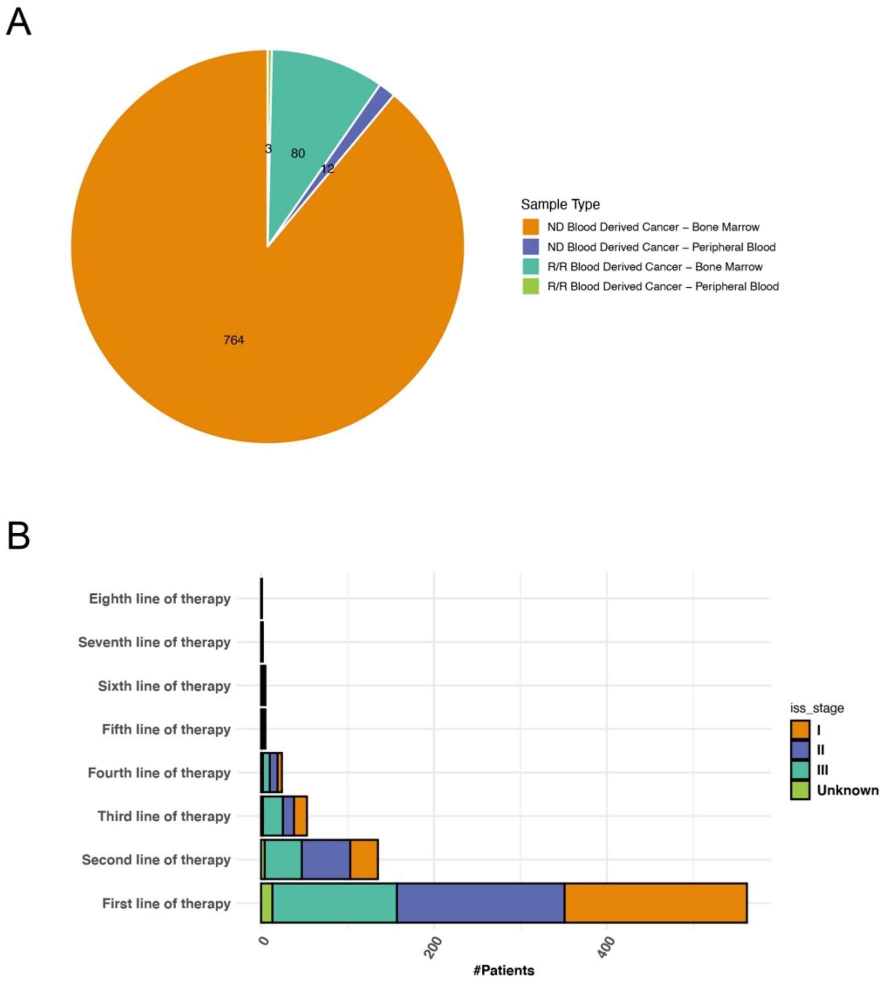

Supplementary Figure 1. Sample information in the original data set. (A) Tissue source of MM samples. Colors indicate different tissue types. (B) Patient line therapy summary. Colors indicate different line therapies.

Figure S2

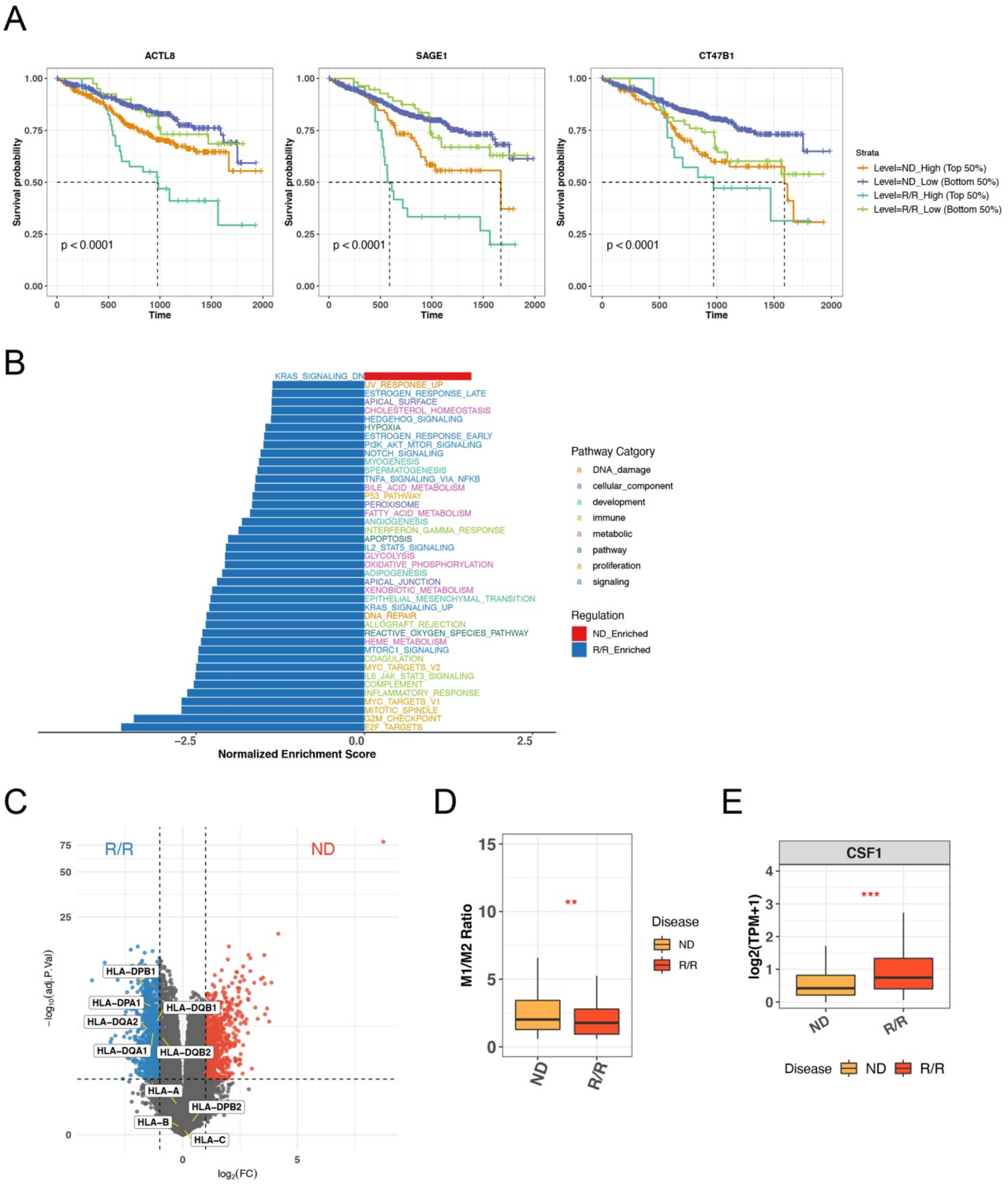

Supplementary Figure 2. Difference between ND and R/R MM tumors. (A) Kaplan Meier survival curve for ACTL8, SAGE1, and CT47B1 expression. (B) Normalized enrichment scores of tumor-up-regulated Hallmark pathways. Text colors indicate different Hallmark pathway categories while bar colors indicate enrichment in ND or R/R tumors. Asterisks denote significance level (*** FDR < 0.001; ** FDR < 0.01; * FDR < 0.05; NS FDR > 0.05). (C) Differential expression of HLA molecules between ND and R/R MM tumors. (D) Ratio of M1 to M2 macrophages maker expression in ND and R/R MM tumors. (E) CSF1 expression in ND and R/R MM tumors. (*** P < 0.001; ** P < 0.01; * P < 0.05; NS P > 0.05).

Figure S3

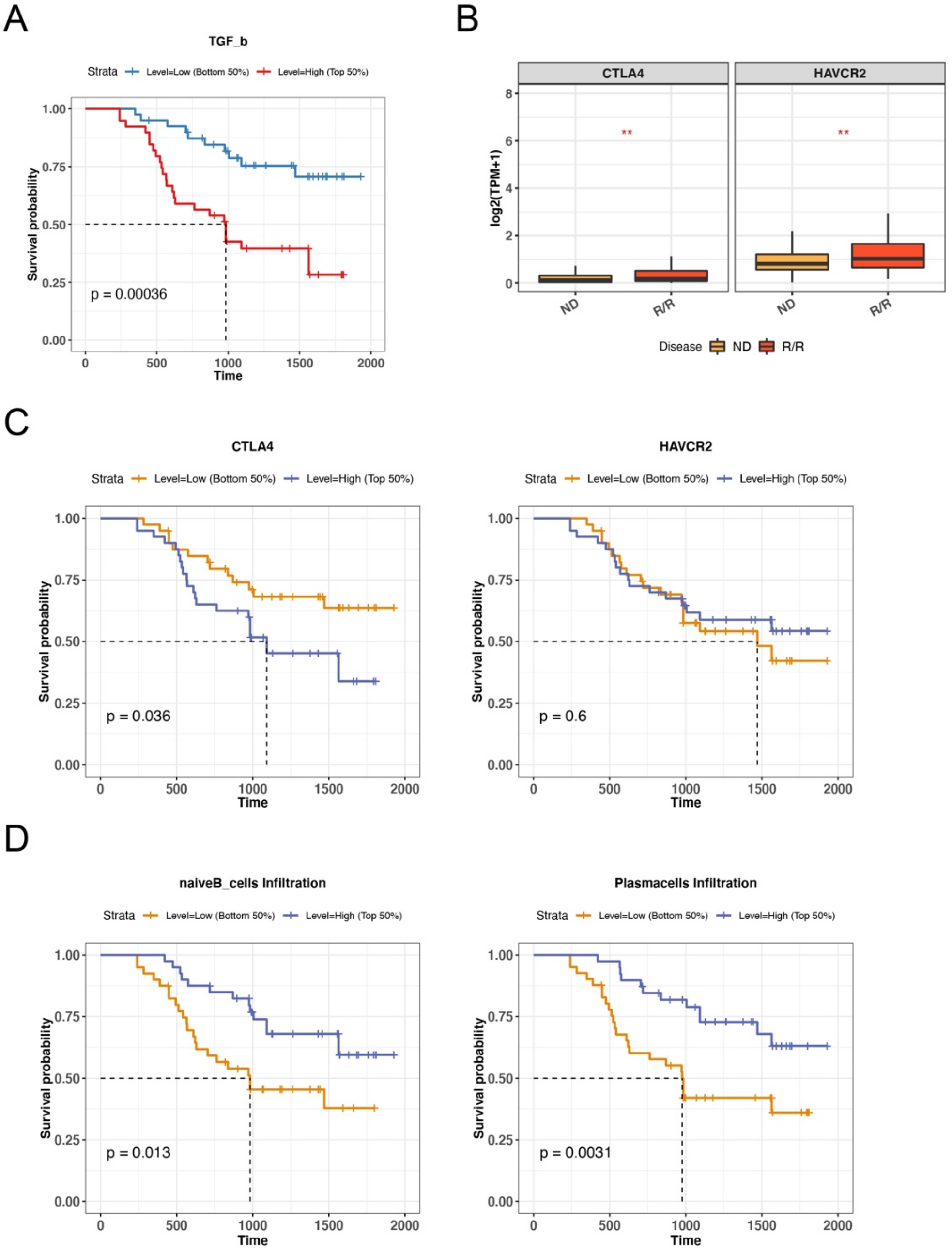

Supplementary Figure 3. Features characterizing R/R MM tumors. (A) Kaplan Meier survival curve for TGF-b immune signature score. (B) CTLA4 and TIM3 expression difference between ND and R/R MM tumors. (C) Kaplan Meier survival curve for CTLA4 and TIM3 expression. (D) Kaplan Meier survival curve for naïve B cell infiltration and normal plasma cell infiltration.

Figure S4

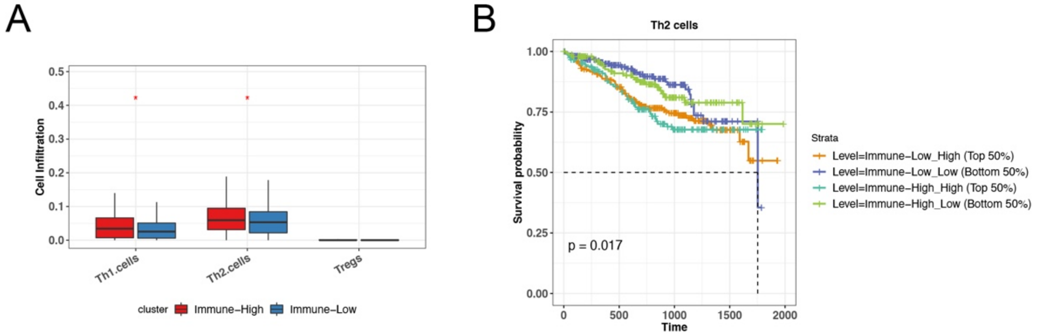

Supplementary Figure 4. Immune clusters difference of ND MM tumors. (A) CD4+ T differentiated cell infiltration difference between immune-low and immune-high tumors, including Th1 cells, Th2 cells, and Th17 cells. (B) Kaplan Meier survival curve for Th2 cell infiltration stratification.

Figure S5

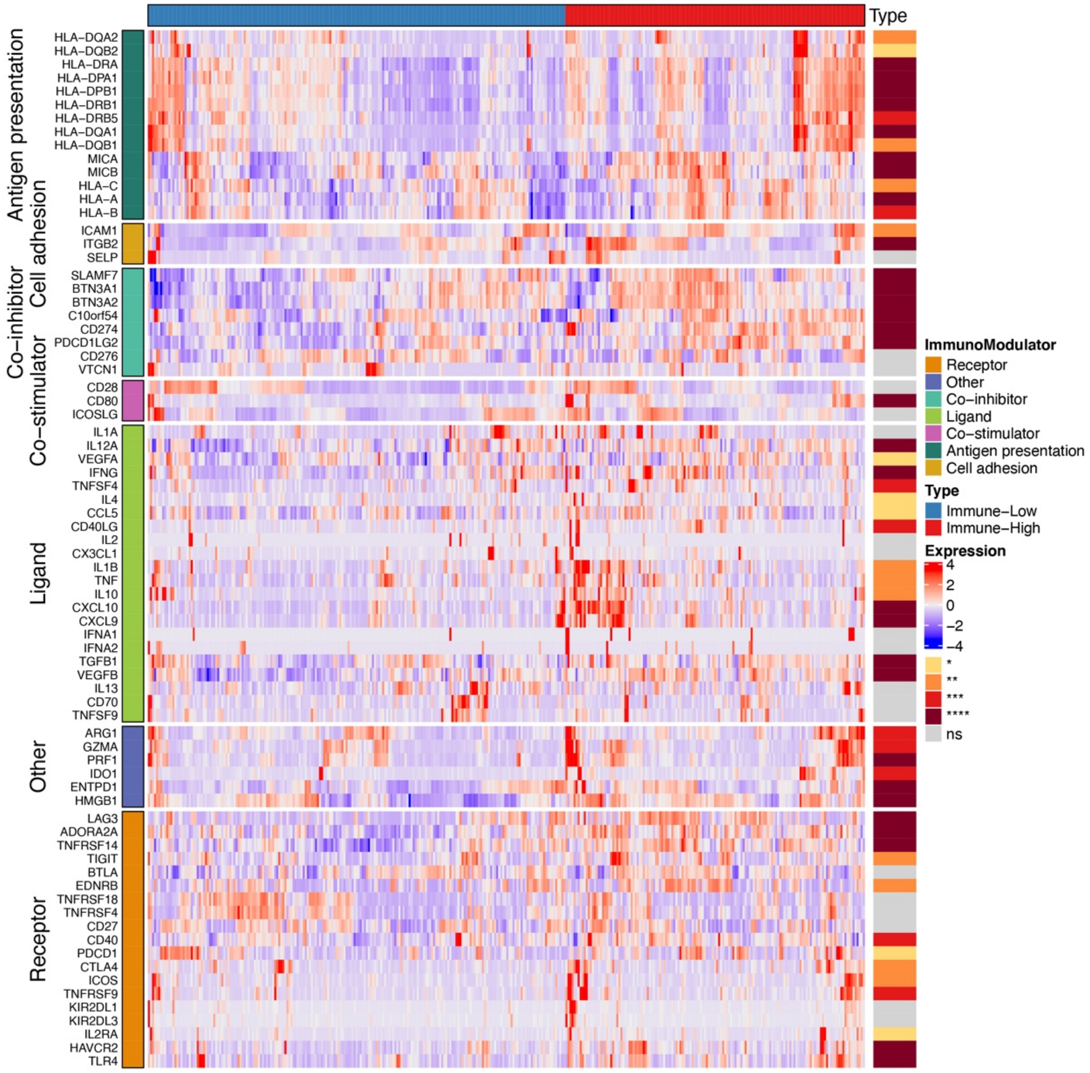

Supplementary Figure 5. IMs expression comparison between immune-low and immune-high of ND MM who are ASCT not eligible. The left bar indicates the categories of IMs. The right bar indicates the significance of expression difference between immune-low and immune-high MM tumors.

Figure S6

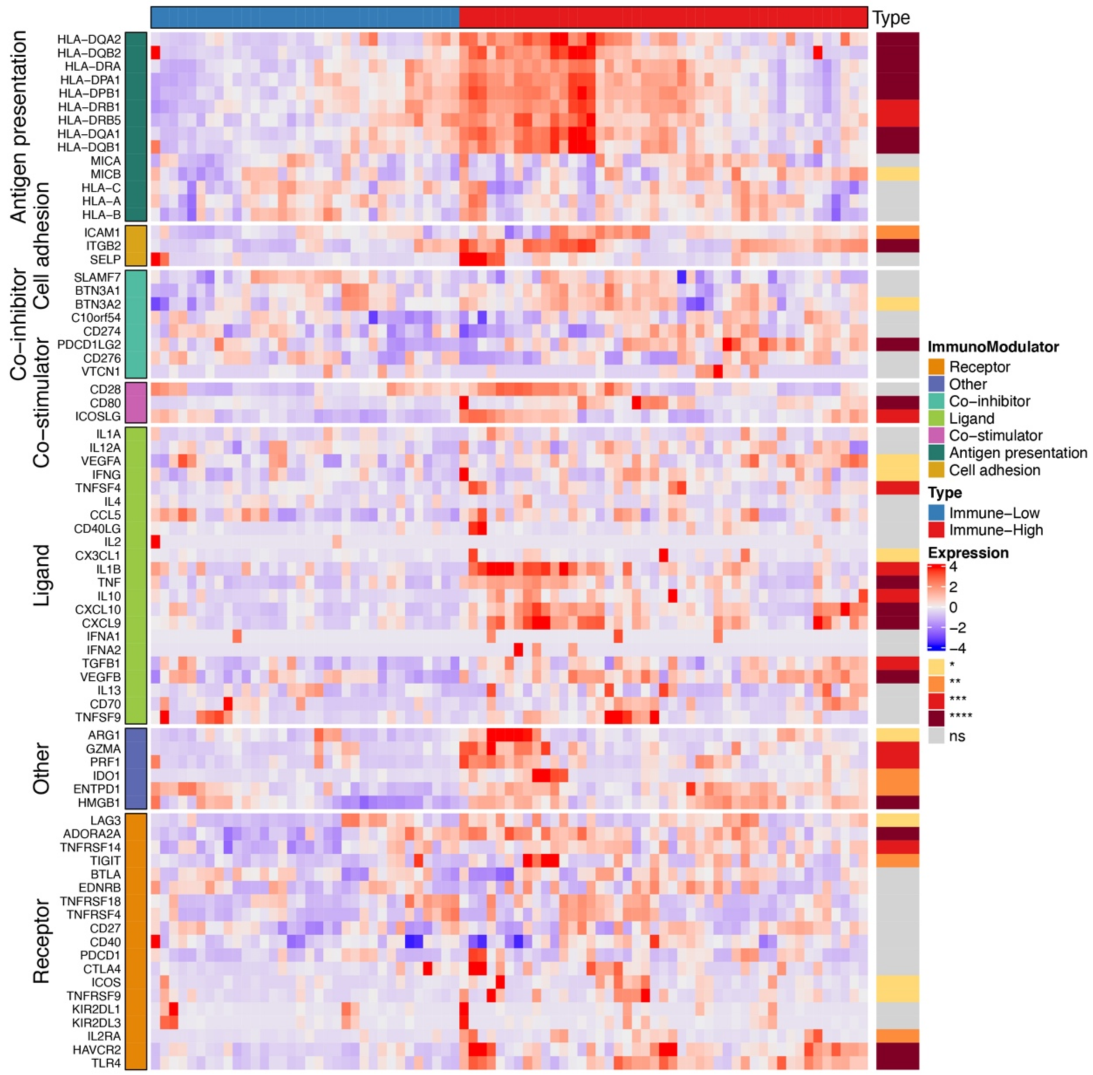

Supplementary Figure 6. IMs expression comparison between immune-low and immune-high of R/R MM who are ASCT not eligible. The left bar indicates the categories of IMs. The right bar indicates the significance of expression difference between immune-low and immune-high MM tumors.
